# An Obstinate Case of Constipation

**Published:** 1893-07

**Authors:** Pryor W. Fitts

**Affiliations:** Mountville, Ga.


### AN OBSTINATE CASE OF CONSTIPATION.

MOUNTVILLE, GA., May 5, 1893.

*Editor Atlanta Medical and Surgical Journal:*

On January 8, 1890, I was called to a male child about four months old that had not had a passage from its bowels in seventeen days. Its parents had given it castor oil several times, but it had failed to produce the desired result.

I found its bowels very much distended and it seemed to be suffering intensely. I gave it a dose of calomel and returned a few hours later only to find it in a worse condition. I used glycerine suppositories on it several times, but did not succeed in getting anything from its bowels but gas. I used an enema of warm water but it did no apparent good. I requested its parents to continue to use the enema occasionally which they did with the result of a copious evacuation of its bowels several hours after I had left.

I found the child doing as well as could have been expected on the following day, but its abdomen remained too large.

It continued to be troubled with constipation and sometimes its bowels were very difficult to move. I gave it various things but found nothing that had any apparent curative effect.

At the age of two years it required as large a dose of medicine to move its bowels as an adult.

Podophyllin had a better effect on it for quite awhile than anything else, but finally it, as well as all other medicines, seemed to lose its effect entirely, and its parents were compelled to resort to enema in order to get its bowels moved. They would fail to get a movement sometimes for two or three weeks, and as a result its abdomen became hard and very much distended, from which it seemed to suffer intense pain.

At the age of three years its abdomen was two or three times as large as it should have been, and its lower ribs were pressed outward almost in a horizontal direction. I was called in at this time to see it. It had been suffering from constipation, but at the time I saw it it had diarrhœa that had followed an enema that had been given it several days before. This was accompanied by severe vomiting. The vomiting ceased however after a few days, but the diarrhœa continued till the child died.

I did not make an autopsy, and, of course, will have to let every man be his own judge as to the cause of the trouble.

PRYOR W. FITTS, M. D.

---

For anæmia and chlorosis I know of nothing that will so soon restore the corpuscles and coloring matter of the blood to a normal standard as the combination of hydrogen dioxide with iron thus:

℞. Tr. ferri chloridi, ʒiij.

Ac. phosphor. dil., ʒv.

Glycerini, ʒij.

Hydrogen dioxide (10 volume solution) ad ℥iv.

M. Sig.—Two drams in water three or four times daily.

—*Solomon Solis-Cohen in Medical News.*

